# Influence of flight pattern on the effectiveness of unmanned aerial vehicles application in a mountain Nanguo pear orchard

**DOI:** 10.3389/fpls.2026.1803212

**Published:** 2026-04-10

**Authors:** Shuang Guo, Jianghui Luo, Hao Yan, Weixiang Yao, Yanhua Meng

**Affiliations:** 1 School of Intelligent Science and Information Engineering, Shenyang University, Shenyang, China; 2 College of Information and Electrical Engineering, Shenyang Agricultural University, National Digital Agriculture Regional Innovation Center (Northeast), Shenyang, China; 3 China Agricultural Technology Extension Association, Beijing, China

**Keywords:** droplet deposition, flight pattern, mountain orchard, Nanguo pear, spray, UAV

## Abstract

**Introduction:**

The application of unmanned aerial vehicles (UAVs) in orchards has been gradually emerging. Due to the complex architecture of tree canopies and the planting environment, choosing a reasonable UAV flight pattern to effectively enhance droplet deposition on critical target areas remains a challenge.

**Methods:**

This study employed Nanguo pear trees as the application target, with an electric multi-rotor UAV, the EA-30X, chosen as the spraying platform. Through comprehensive droplet assessment methodologies, five different flight patterns (intra-row, intra-row-high-speed, intra-row-half-rate, inter-row, verti-row) were analyzed and compared to assess droplet deposition in the tree canopy.

**Results:**

Measurements revealed that 71.85% of the droplet coverage is in the 0-5% range and the droplet density is in the 0-200 drops·cm^-^² range. The results also showed that there was no statistically significant difference in droplet deposition between the inner and outer zones of the fruit tree canopy in the horizontal direction among the treatments (p > 0.05).

**Discussion:**

The results indicate that, under the conditions of constant spray volume rate (60 L/ha) and flight height (2.5 m), particularly when natural wind speeds are excessive, using a UAV for two-pass spraying patterns (intra-row-high-speed, intra-row-half-rate) is not recommended. Intra-row, inter-row and verti-row are viable options, but the selection should be made flexibly based on operational requirements. Different flight patterns lead to changes in the droplet deposition distribution trends across vertical layers and between inner and outer zones. This study provides scientific and precise operational guidance and reference for pest and disease control in Nanguo pear orchards.

## 1 Introduction

The unmanned aerial vehicle (UAV) spraying technology can be traced back to the 1980s (Guebsi et al., 2024). Since the beginning of the 21st century, the rapid development of technology particularly breakthroughs in sensors, GPS positioning, and autonomous navigation has led to significant improvements in UAV-based pesticide application technology and facilitated its gradual adoption (Zhang et al., 2023; Gatkal et al., 2025). Especially in China, UAVs have become increasingly popular in plant protection due to relatively relaxed regulatory policies. In the early stage, UAVs were primarily utilized for field crops such as rice (Paul et al., 2024; Wongsuk et al., 2024), wheat (Dammer et al., 2022), maize (Wang et al., 2023), and soybeans (Hiremath et al., 2024), where large planting areas and relatively open environments provided ideal operating conditions for aerial spraying. As the technology matured and market saturation grew, UAV manufacturers have gradually shifted their focus toward orchard spraying applications, which involve more complex spatial structures and operating environments (Zhang et al., 2021; Wei et al., 2022).

In China, arable land resources are scarce, and a considerable proportion of orchards are therefore distributed in mountainous and hilly regions to conserve flat farmland for grain production (Shen et al., 2024). Although this strategy maximizes land-use efficiency, it also poses significant challenges for orchard management. Most mountainous orchards are non-standardized, lacking pathways for ground-based spraying machinery, and the steep terrain further hinders pesticide application operations (Cui et al., 2025). Under this backdrop, UAV-based spraying technology demonstrates its unique advantages. UAV flight is less constrained by terrain, does not require dedicated takeoff and landing sites, and enables highly efficient spraying while reducing labor input and eliminating direct exposure risks to operators (Godyń et al., 2025; Papadogiorgou et al., 2025; Yao et al., 2025). Consequently, the application of UAVs in orchards has gradually emerged and has gained growing public acceptance.

To enhance the applicability of UAVs in orchards, manufacturers have implemented a series of improvements. In orchards with undulating terrain, UAVs can perform intelligent terrain-following flight, whereby the flight altitude is automatically adjusted according to topographic variations to maintain a constant spraying distance from the tree canopy. UAVs can also automatically avoid obstacles in orchards. Representative manufacturers and models include Eavision Technologies Co., Ltd., (Suzhou, China) with the EA-30X and EA-J100, and DJI Technology Co., Ltd., (Shenzhen, China) with the T50, T60, and T100. Eavision’s UAVs are equipped with a binocular vision 3D perception system, which integrates with millimeter-wave radar and LiDAR technologies, eliminating the need for pre-mapping. During operation, the system detects terrain and obstacles in real time and allows for operation day or night. Moreover, its proprietary Constant-Temperature Mist Spray (CCMS) system allows adjustable droplet size and precise flow-rate control, offering an effective technical solution to the droplet penetration problem in three-dimensional (3D) crops such as fruit trees. DJI has also been extensively applied in orchard spraying. The radar system integrated with a vision module enables accurate mapping of complex orchard terrain and obstacles, facilitating fully automated terrain-following operations. Based on the distribution of fruit trees, precise three-dimensional spraying flight paths can be generated automatically. In addition, the dual centrifugal atomization nozzles can also enhance droplet penetration within tree canopies.

Unlike annual dense crops such as rice and wheat, which consist of small individual plants that are uniformly distributed with minimal gaps within the planting area, fruit trees are characterized by large canopy sizes and distinct gaps between rows and between individual trees. Consequently, higher requirements are imposed on UAV flight path planning in orchards (Tang et al., 2018). How to select appropriate UAV flight patterns to effectively enhance droplet deposition on critical target sites within fruit tree canopies has become a research hotspot in recent years.

In current studies on UAV-based orchard spraying, a clear diversification of UAV flight patterns has been observed. Most studies have adopted the flight pattern in which the UAV flies along the tree rows directly above the canopy (Martinez-Guanter et al., 2020; Liu et al., 2020; Jiang et al., 2022; Yan et al., 2023). Some studies have employed flight paths perpendicular to the tree rows (Sarri et al., 2019; Wang et al., 2021), while others have used routes parallel to the rows but between adjacent tree rows (Chen et al., 2020; Meng et al., 2022). Additionally, in the study by Li et al. (2021), to achieve an application rate of 93.6 L/ha, a pattern of spraying twice at 46.8 L/ha per pass was implemented. Meng et al. (2020) investigated droplet deposition in peach orchards with different tree architectures under various UAV flight routes (intra-row vs inter-row) and numbers of spray times (1 vs 2). The results indicated that for Y-shape peach, intra-row route obtained a higher droplet coverage rate, while for Central Leader-shape peach, inter-row resulted in a higher droplet coverage rate. This study ultimately recommended “spraying once” in practical applications for Y-shaped peach trees to achieve optimal efficacy. Wang et al. (2022) compared droplet deposition in apple orchards under three UAV flight patterns, namely intra-row, inter-row, and verti-row, and found that the effect of different flight patterns on the distribution of spray deposition within the canopy was relatively limited. Qi et al. (2023) conducted spraying operations in pear orchards using two UAVs, one flying directly above the tree rows (along the rows) and the other flying between rows along the row direction; however, no direct comparison between the two patterns was reported. The study by Psiroukis et al. (2026) on UAV spraying in vineyards showed that over-row treatments led to concentrated deposition in the upper canopy. In contrast, inter-row treatments produced more uniform deposition profiles but were more susceptible to wind direction. Biglia et al. (2022) evaluated droplet deposition in vineyards under three flight patterns: one-way flights across the vine rows, round-trip flights across the vine rows, and a single pass precisely above the vine row. The results showed that the two patterns perpendicular to the rows were impractical, with very low measured canopy deposition, whereas spraying along the rows maximized canopy deposition and minimized off-target losses. In contrast, Sarri et al. (2019) reported preliminary results for vineyards indicating insufficient spray coverage when flying along the rows, with most droplets depositing in the center between rows. Therefore, the flight pattern perpendicular to the rows was adopted.

These studies reveal the complexity of UAV-based spraying operations in orchards, indicating that different crop planting patterns and canopy structural characteristics substantially influence operational strategies such as flight path planning and the number of spray times. Therefore, it is necessary to conduct targeted experiments for different orchard types and operating environments. Nanguo pear is a characteristic pear cultivar in Liaoning Province in northeastern China, where it is mainly cultivated in mountainous areas with complex terrain and poor accessibility. Owing to topographic constraints, pest and disease control in Nanguo pear orchards was previously dominated by manual spraying using knapsack sprayers. Such manual spraying is not only inefficient but also poses a risk of pesticide poisoning to operators. Since 2021, we have been exploring the applicability of UAV-based spraying in Nanguo pear orchards. Focusing on the spot-spray operation mode of the DJI T20, the effects of nozzle type, spray volume rate, and adjuvants on droplet deposition characteristics were investigated (Guo et al., 2022). However, due to the low operational efficiency of the spot-spray, which is insufficient for large-area spraying, subsequent studies have been conducted based on continuous flight patterns. For UAV spraying applications in mountainous Nanguo pear orchards, we have proposed a spray-swath determination method suitable for such environments (Liu et al., 2024), comparatively analyzed droplet deposition differences among trees with different canopy sizes (Guo et al., 2024), and further investigated droplet deposition under a pre-wetting application strategy (Yao et al., 2026). These studies have preliminarily confirmed the feasibility of UAV spraying in Nanguo pear orchards; however, the effects of different flight patterns on droplet deposition characteristics in these orchards remain to be further clarified.

Therefore, based on preliminary research into the application of aerial spraying in mountainous orchards, this study selected an electric multi-rotor UAV as the spraying platform. The objective is to comprehensively compare multiple UAV flight patterns in mountain Nanguo pear orchards, aiming to explore the possible differences in droplet deposition. Under the condition that the overall spray volume rate in the experimental area was kept constant, this study systematically analyzed and compared droplet deposition within the canopies of Nanguo pear trees under five distinct UAV flight patterns, thereby partially filling the gap in research on flight patterns in mountainous orchards. At the same time, the results also help operators make decisions on operational strategies based on actual conditions, providing more scientific and precise guidance for pest and disease control in mountainous Nanguo pear orchards, and supporting the selection of the optimal flight pattern according to specific orchard conditions and the spatial distribution of pests and diseases.

## 2 Materials and methods

### 2.1 Experimental site

The experiment was conducted in a commercial Nanguo pear orchard located in Haicheng City, Liaoning Province, China (122°56′47″E, 40°52′3″N), on a slope of approximately 20°. Within the experimental area, the average tree height was 3.1 m, and the age of the trees was 15 years, with both row spacing and tree spacing at 5.0 m. The tree form is a three-main-branch layered structure, with an average canopy dimensions were 4.5×4.7×2.3 m (diameter along row × diameter across row × height).

### 2.2 Experimental equipment

**Figure 1:**
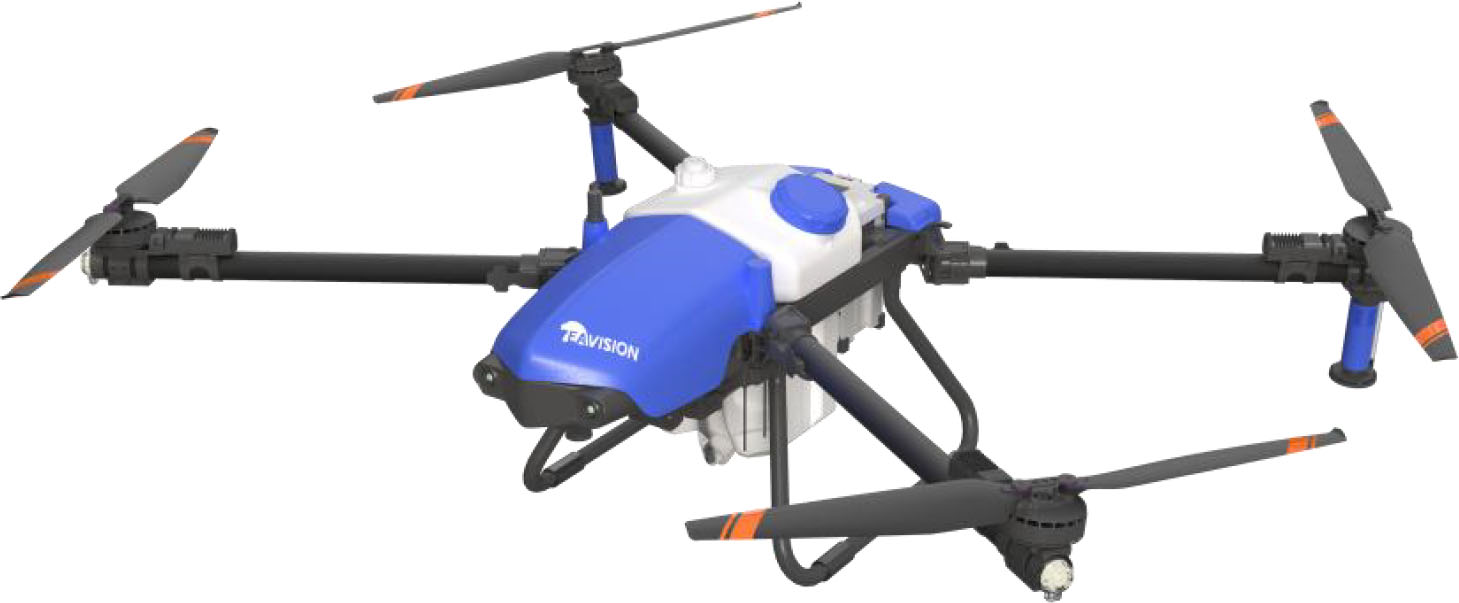
EA-30X UAV.

As shown in **Figure 1**, an EA-30X UAV (Eavision Technologies Co., Ltd., Suzhou, China) was used for spraying operations. The UAV has a maximum wheelbase of 2200 mm, unfolded dimensions of 2350 × 2760 × 620 mm, and a maximum takeoff weight of 67 kg. The spraying system consists of a tank with a capacity of 30 L, a diaphragm pump that can automatically adjust the spraying flow rate, an ultrasonic flowmeter with a flow measurement error of less than 5%, and two CCMS-L20000 (Constant-Temperature Mist Spray) ambient temperature mist nozzles located directly under the rear rotor, enabling spray droplet size control within the 20-250 μm range and delivering an effective swath width of 7–8 m.

### 2.3 Experimental design

**Figure 2:**
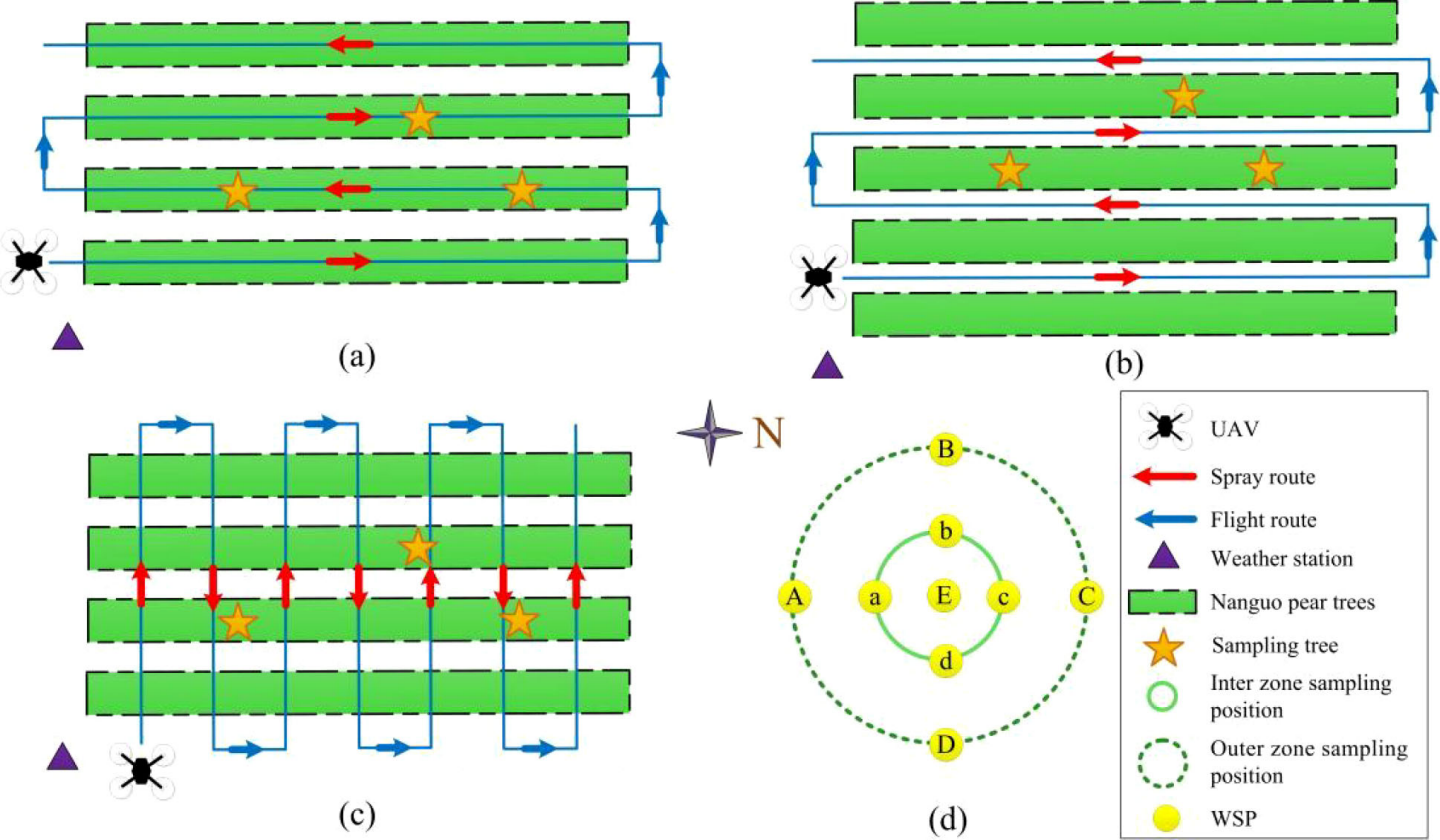
Schematic diagram of flight patterns and droplet sampling: **(a)** intra-row, intra-row-high-speed, and intra-row-half-rate; **(b)** inter-row; **(c)** verti-row; **(d)** layout of droplet sampling points. Drawing was not to scale.

**Figure 3:**
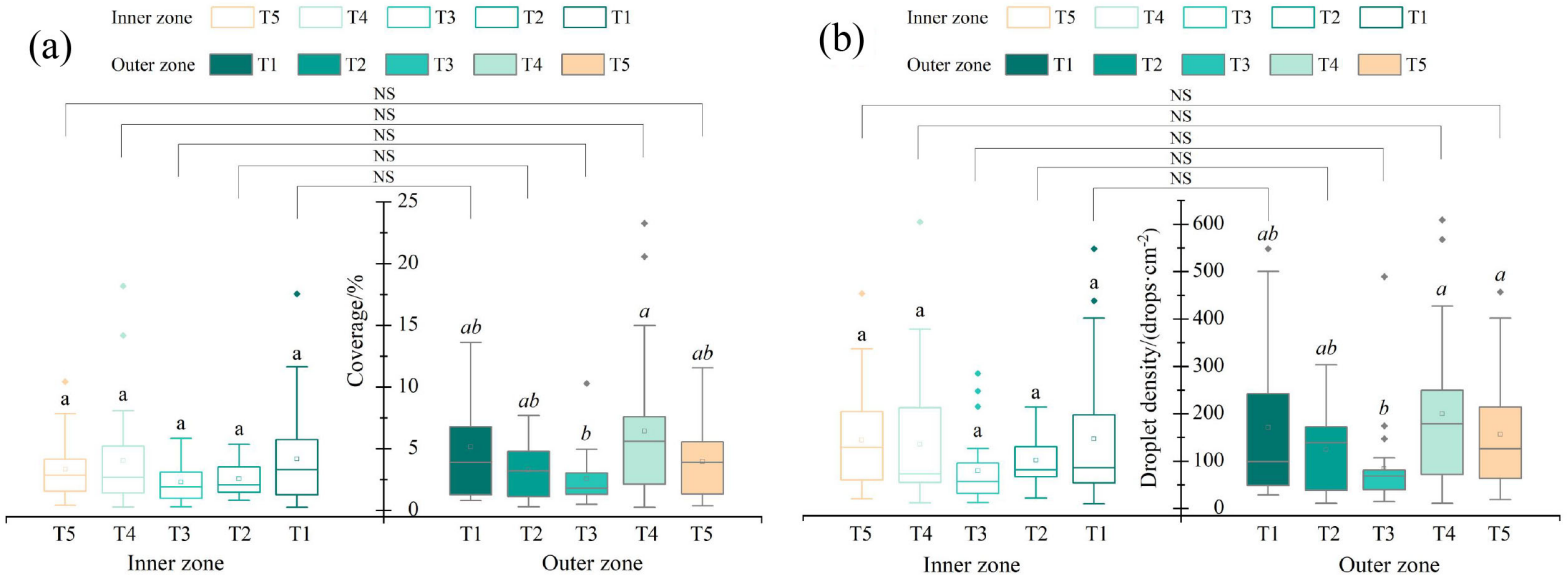
**(a)** Droplet coverage and **(b)** droplet density in the inner and outer zones of the tree canopy under different flight patterns. Upright and italic letters indicate the results of significance analysis for the inner and outer zones, respectively, and the same letters denote no statistically significant difference. NS indicates no significant difference between the inner and outer zones within the same treatment.

**Figure 4:**
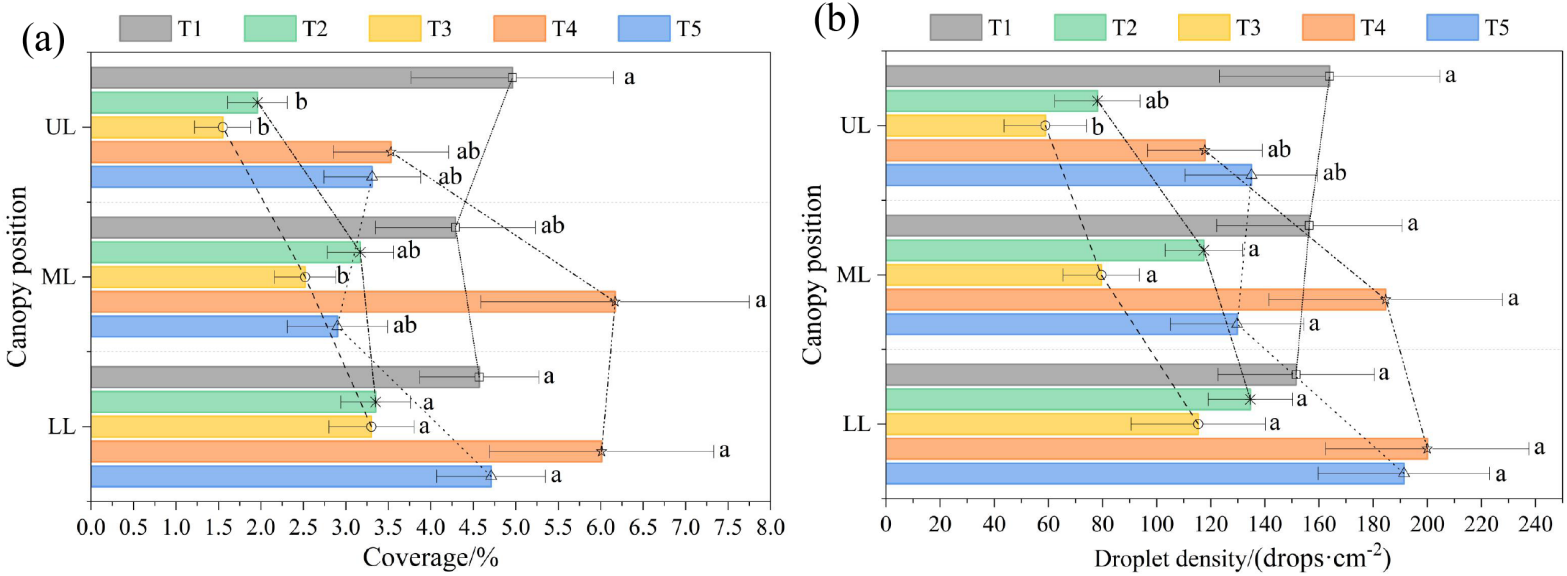
Distribution of **(a)** droplet coverage and **(b)** droplet density in the vertical direction of the tree canopy for each treatment. UL, Upper layer; ML, Middle layer; LL, Lower layer.

To assess the effects of UAV flight patterns on droplet deposition in Nanguo pear orchards, the field experiment comprised five treatments (T1-T5), with detailed operational parameters listed in **Table 1**, each corresponding to a specific flight pattern. The five flight patterns were: (1) the flight path was directly above the tree rows, and the UAV performed a single spray pass along the row direction (named intra-row, **Figure 2a**); (2) the flight path was directly above the tree rows, and the UAV conducted two spray passes along the rows, with the flight speed doubled relative to pattern (1) (named intra-row-high-speed, **Figure 2a**); (3) the flight path was directly above the tree rows, and the UAV conducted two spray passes along the rows, with the spray volume rate reduced by half compared with pattern (1) (named intra-row-half-rate, **Figure 2a**); (4) the flight path was directly above the inter-row space, and the UAV flew parallel to the rows to perform a single spray pass (named inter-row, **Figure 2b**); and (5) the flight path was perpendicular to the tree rows, and the UAV flew once along the slope direction (named verti-row, **Figure 2c**). To minimize confounding factors and ensure accurate comparison among flight patterns, the operational height for all treatments was set to 2.5 m above the top of the tree canopy and the flight speed was fixed at 2 m/s (except the intra-row-high-speed) based on previous operational experience. In addition, clean water was used as the spraying agent to facilitate repeated trials, and each treatment was replicated three times.

**Table 1:** Experiment treatment setup.

Treatment	Flight pattern	Spraying volume rate (L/ha)	Spray times	Flight height (m)	Flight speed (m/s)
T1	Intra-row	60	1	2.5	2
T2	Intra-row-high-speed	60	2	2.5	4
T3	Intra-row-half-rate	30*2	2	2.5	2
T4	Inter-row	60	1	2.5	2
T5	Verti-row	60	1	2.5	2

30*2 indicated that T3 involved two spraying operations, each applied at a spray volume rate of 30 L/ha.

Three Nanguo pear trees were randomly selected in the experimental area for droplet sampling to evaluate the droplet deposition quality. Referring to the research by Guo et al. (2022) and Liu et al. (2024), the canopy area of each tree was divided into three sampling layers (upper, middle, and lower layer) at heights of 2.5 m (upper layer), 1.8 m (middle layer), and 0.9 m (lower layer) above ground. Each sampling layer comprised nine droplet sampling points (**Figure 2d**). Specifically, sampling points A, B, C, and D were located in the outer zone of the canopy, sampling points a, b, c, and d were located in the inner zone of the canopy, and point E was located in the center of the canopy. The sampling diameters of the outer zone were 2.0 m (upper layer), 3.5 m (middle layer), and 2.8 m (lower layer), respectively, and the sampling diameters of the inner zone were 1.1 m (upper layer), 2 m (middle layer), and 1.5 m (lower layer), respectively. Water-sensitive papers (WSP, 26×76 mm, Syngenta Crop Protection AG, Basel, Switzerland) were used as droplet collectors. To ensure measurement accuracy, two pieces of WSPs were attached to both the adaxial and abaxial surfaces of a leaf at each sampling point along the midrib using clips.

During each treatment, meteorological data (wind speed, wind direction, air temperature, and relative humidity) were recorded using a Kestrel 5500 Link portable weather station (Nielsen-Kellerman, Minneapolis, MN, USA). The weather station was positioned 15 m from the experimental area at a height of 3 m above the ground. All data were logged at 2 s intervals throughout the experiments.

### 2.4 Data analysis

After each treatment, the WSPs were labeled and stored in a cool and dry place. In the laboratory, the collected WSPs were scanned into grayscale images using a scanner at a resolution of 600 dpi, then the WSP images were processed using DepositScan software (Zhu et al., 2011) to determine deposition data.It should be noted that the droplet coverage or droplet density at each sampling point was calculated by summing the values measured on the adaxial and abaxial surfaces of the leaf at that point.

To evaluate horizontal droplet deposition within the canopy, the canopy inner–outer deposition coefficient (C_IO_), a comprehensive deposition coefficient (C_D_) which integrates data from both along row and perpendicular to the row directions, and the coefficient of variation of deposition uniformity across all sampling points within the layer (CV-U) were calculated. In addition, the coefficient of variation was also used to characterize vertical droplet penetration among different canopy layers (CV-P). A lower CV value indicates better deposition uniformity or penetration performance.

The C_IO_ was calculated using the following equation, according to Equation 1:

(1) $$C_{IO}=\frac{D_{inner}}{D_{outer}}$$

where $D_{inner}$ is the mean droplet coverage or droplet density of the inner canopy zone, and $D_{outer}$ is the corresponding mean value of the outer canopy zone. A C_IO_ value closer to 1 indicates a more uniform deposition between the inner and outer canopy zones.

Within each canopy layer, the direction parallel to the tree rows was defined as the X direction, comprising sampling points A, a, E, c, and C. The direction perpendicular to the tree rows was defined as the Y direction, comprising points B, b, E, d, and D. The C_D_ was calculated using the following equation, according to Equation 2:

(2) $$C_{D}=(\frac{D_{X}}{D_{Y}}-1)$$

where $D_{X}$ denotes the mean droplet coverage or droplet density in the X direction and $D_{Y}$ denotes the corresponding mean value in the Y direction. A positive C_D_ value indicates higher deposition in the X direction, whereas a negative value indicates higher deposition in the Y direction. The closer the absolute value of C_D_ is to zero, the more uniform the droplet deposition between the two directions.

the CV was calculated using the following equation, according to Equations 3, 4:

(3) $$CV=\frac{S}{\bar{X}}$$

(4) $$S\sqrt{\frac{\sum_{i=1}^{n}{\left(X_{i}-\bar{X}\right)}^{2}}{n-1}}$$

where S is the standard deviation, *X_i_* refers to the coverage or deposition density at sampling point *i*, $\bar{X}$ denotes the average deposition value at all sampling points, and *n* is the number of sampling points.

All statistical analyses were performed using IBM SPSS Statistics (IBM Corp., Armonk, NY, USA). Normality test and homogeneity of variance test were performed before data analysis. One-way analysis of variance (ANOVA) was applied to compare droplet deposition between the inner and outer canopy zones. Least significant difference (LSD) tests were employed for multiple comparisons of droplet deposition differences between treatments and and across the upper, middle, and lower canopy layers at significance level 0.05.

## 3 Results

### 3.1 Meteorological conditions

The meteorological conditions for each treatment during the experiments are presented in **Table 2**. Ambient temperature and relative humidity remained relatively stable throughout the trials, with mean values of 28.9 °C and 73.7%, respectively. Wind speed was 1.3 m/s for T2 and ranged between 0.2-0.8 m/s for other treatments. Although wind direction varied, southerly winds were predominant.

**Table 2:** Summary of meteorological data for each treatment.

Treatment	Temperature/°C	Humidity/%	Wind velocity/(m·s^-1^)	Wind direction
T1	29.0 ± 0.7	75.1 ± 1.6	0.8 ± 0.4	S
T2	29.4 ± 0.7	72.5 ± 1.4	1.3 ± 0.5	S
T3	29.2 ± 0.7	71.1 ± 2.7	0.3 ± 0.4	SW
T4	28.8 ± 0.3	79.6 ± 4.6	0.4 ± 0.4	S
T5	28.2 ± 0.6	70.0 ± 2.1	0.2 ± 0.3	SE

The values are presented as mean value ± standard error.

### 3.2 Horizontal droplet deposition within the tree canopy

The droplet coverage in the inner and outer canopy zones for each treatment in the horizontal direction is illustrated in **Figure 3a**. In the inner zone, the mean droplet coverage of T1 and T4 exceeded 4%, reaching 4.18% and 4.03%, respectively, followed by T5 with a coverage of 3.36%, whereas T2 and T3 exhibited the lowest values, both below 3%. Although numerical differences were observed, no significant differences were detected among treatments in the inner zone (p > 0.05). In the outer zone, T4 and T1 again showed the highest mean droplet coverage, at 6.44% and 5.77%, respectively. In contrast, T3 exhibited the lowest coverage, only 2.58%, which was significantly lower than that of T4, and a significant difference was observed between these two treatments (p< 0.05).

The trend of droplet density was similar to that of droplet coverage (**Figure 3b**). In the inner zone, the maximum droplet density was 147.3 drops·cm^-^² for T1, whereas the minimum value was 79.9 drops·cm^-^² for T3; no significant differences were observed among treatments (p > 0.05). In the outer zone, the droplet densities of T4 (200.2 drops·cm^-^²) and T5 (156.8 drops·cm^-^²) were significantly higher than that of T3 (84.3 drops·cm^-^²), showing significant differences (p< 0.05), whereas differences among the remaining treatments were not significant (p > 0.05). These results indicate that changes in flight pattern had limited effects on droplet deposition in the inner canopy zone but exerted a noticeable influence on deposition in the outer zone.

In addition, statistical analyses were conducted to compare the inner and outer canopy zones in the horizontal direction within each treatment. Although both droplet coverage and droplet density in the inner zone were numerically lower than those in the outer zone, the p-values for droplet coverage were 0.386 (T1), 0.381 (T2), 0.680 (T3), 0.062 (T4), and 0.477 (T5), indicating no significant differences between the inner and outer zones within any treatment. Similarly, for droplet density, the corresponding p-values were 0.557 (T1), 0.353 (T2), 0.680 (T3), 0.120 (T4), and 0.726 (T5), also showing no significant differences between the inner and outer canopy zones for any treatment.

### 3.3 Vertical droplet deposition within the tree canopy

The vertical distribution of droplet deposition within the tree canopy is illustrated in **Figure 4**. For droplet coverage, T1 exhibited the highest value in the upper canopy layer (4.96%), whereas T3 showed the lowest coverage (1.55%). A significant difference was observed between T3 and T5 in the upper layer (p< 0.05). In the middle and lower canopy layers, T4 achieved the highest droplet coverage, reaching 6.17% (middle layer) and 6.01% (lower layer), respectively, while T3 remained the lowest, with values of 2.52% (middle layer) and 3.30% (lower layer). Furthermore, although numerical differences in coverage were observed among treatments in the middle and lower layers, no statistically significant differences were detected (p > 0.05).

Further analysis of the vertical droplet deposition trend for each treatment revealed that T1 achieved the highest droplet coverage in the upper canopy layer, with values in the middle and lower layers showing a decreasing trend; however, the differences among the three layers were minimal, with a range of only 0.67%. For both T2 and T3, the upper layer exhibited the lowest coverage (1.96% for T2 and 1.55% for T3), whereas the lower layer showed the highest values (3.35% for T2 and 3.30% for T3), indicating an increasing trend from the upper to the lower canopy. T4 also presented the lowest coverage in the upper layer (3.53%), but the middle and lower layers both exceeded 6%, reaching 6.17% and 6.01%, respectively. This treatment exhibited the largest range among all treatments, at 2.64%. For T5, the maximum coverage (4.71%) occurred in the lower layer, followed by the upper layer (3.32%), with the middle layer showing the lowest value (2.90%), forming a U-shaped curve trend.

For droplet density, the droplet density for each treatment ranged from 58.85 to 163.87 drops·cm^-^² in the upper layer, 79.53 to 184.61 drops·cm^-^² in the middle layer, and 115.36 to 199.98 drops·cm^-^² in the lower layer. T3 exhibited the lowest droplet density across all layers among the treatments. Overall, the distribution trend of droplet density was similar to that of droplet coverage. A notable difference was that for T1, the droplet density in the tree canopy decreased from the upper to the lower layers, whereas for T4, the droplet density increased from the upper to the lower layers.

### 3.4 Droplet deposition evaluation

Further evaluation and analysis of droplet deposition within the canopy for each treatment are summarized in **Table 3**. For the differences in droplet deposition between the inner and outer canopy zones, statistical results indicated that for 80% of the sampling layers, the C_IO_ values for both coverage and density were less than 1, only certain layers of some treatments had C_IO_ values greater than 1. Overall, droplet deposition in the inner canopy zone was lower than in the outer zone. The minimum and maximum C_IO_ values for droplet coverage were found in the middle layer of T2 canopy (0.52) and the upper layer of T3 canopy (1.17), respectively. For droplet density, the minimum and maximum C_IO_ values were found in the middle layer of T4 canopy (0.64) and the upper layer of T3 canopy (1.45), respectively.

**Table 3:** Summary of droplet deposition evaluation indexes for all treatment.

Treatment	Sampling position	C_IO_		C_D_		CV-U		CV-P	
		Coverage	Droplet density	Coverage	Droplet density	Coverage	Droplet density	Coverage	Droplet density
T1	UL	0.83	0.83	-0.03	0.00	104.84%	108.56%	7.34%	3.95%
	ML	0.79	0.90	0.17	0.11	95.33%	95.63%		
	LL	0.81	0.85	-0.03	-0.03	67.10%	83.19%		
T2	UL	0.88	0.70	0.42	0.27	77.45%	88.22%	26.77%	26.33%
	ML	0.52	0.73	0.08	-0.02	53.04%	53.31%		
	LL	1.06	1.01	0.12	0.13	54.01%	50.59%		
T3	UL	1.17	1.45	0.28	0.40	92.14%	112.73%	35.85%	33.08%
	ML	0.91	1.04	0.62	0.64	61.64%	77.37%		
	LL	0.77	0.72	0.05	0.46	66.54%	93.83%		
T4	UL	0.58	0.64	0.93	0.83	83.75%	78.49%	28.32%	26.08%
	ML	0.57	0.58	0.45	0.27	111.69%	102.17%		
	LL	0.71	0.79	0.88	0.54	95.92%	82.01%		
T5	UL	0.89	1.26	0.24	0.23	75.51%	79.26%	26.16%	22.46%
	ML	0.79	0.76	1.17	0.93	88.99%	83.17%		
	LL	0.84	0.83	0.02	-0.01	59.41%	72.22%		

For the differences in droplet deposition between the tree row-parallel direction (X direction) and the tree row-perpendicular direction (Y direction) within the canopy, it is evident that overall, droplet deposition in the X direction is higher than in the Y direction (C_D_ > 0). The maximum C_D_ values for both droplet coverage and droplet density were found in the middle layer of the T5 canopy, with values of 1.17 and 0.93, respectively. This indicates that the largest difference in droplet deposition between the X and Y directions occurred at the middle layer sampling location of T5, where the droplet coverage and droplet density in the X direction were 2.17 times and 1.93 times greater than those in the Y direction, respectively. In contrast, the smallest difference was observed in T1, particularly in the upper canopy layer, where the droplet density C_D_ value was 0, indicating that there was almost no difference in droplet deposition between the X and Y directions at this location.

For the deposition uniformity between sampling points within the same canopy layer, the CV-U values for both droplet coverage and droplet density were generally high, exceeding 50%, with some sampling layers showing CV-U values greater than 110%. This indicates that the actual deposition uniformity of droplets within the canopy is less than ideal, with significant deposition variations between individual sampling points. Compared to other treatments, T2 exhibited relatively better droplet deposition uniformity across all layers, with CV-U values for coverage ranging from 53.04% to 77.45%, and for droplet density, the CV-U values ranged from 50.59% to 88.22%.

Regarding the droplet penetration within the vertical canopy layers of each treatment, T1 exhibited the best droplet penetration, with CV-P values for coverage and droplet density of 7.34% and 3.95%, respectively. These results align with the trends identified in Section 3.3. The CV-P values for T2 to T5 were relatively similar, with the CV-P for coverage ranging from 26.16% to 35.85% and for droplet density ranging from 22.46% to 33.08%. Among these, the highest CV-P values were observed for T3, indicating that T3 exhibited the poorest droplet penetration across all treatments.

### 3.5 Droplet distribution statistics at sampling points

**Figure 5:**
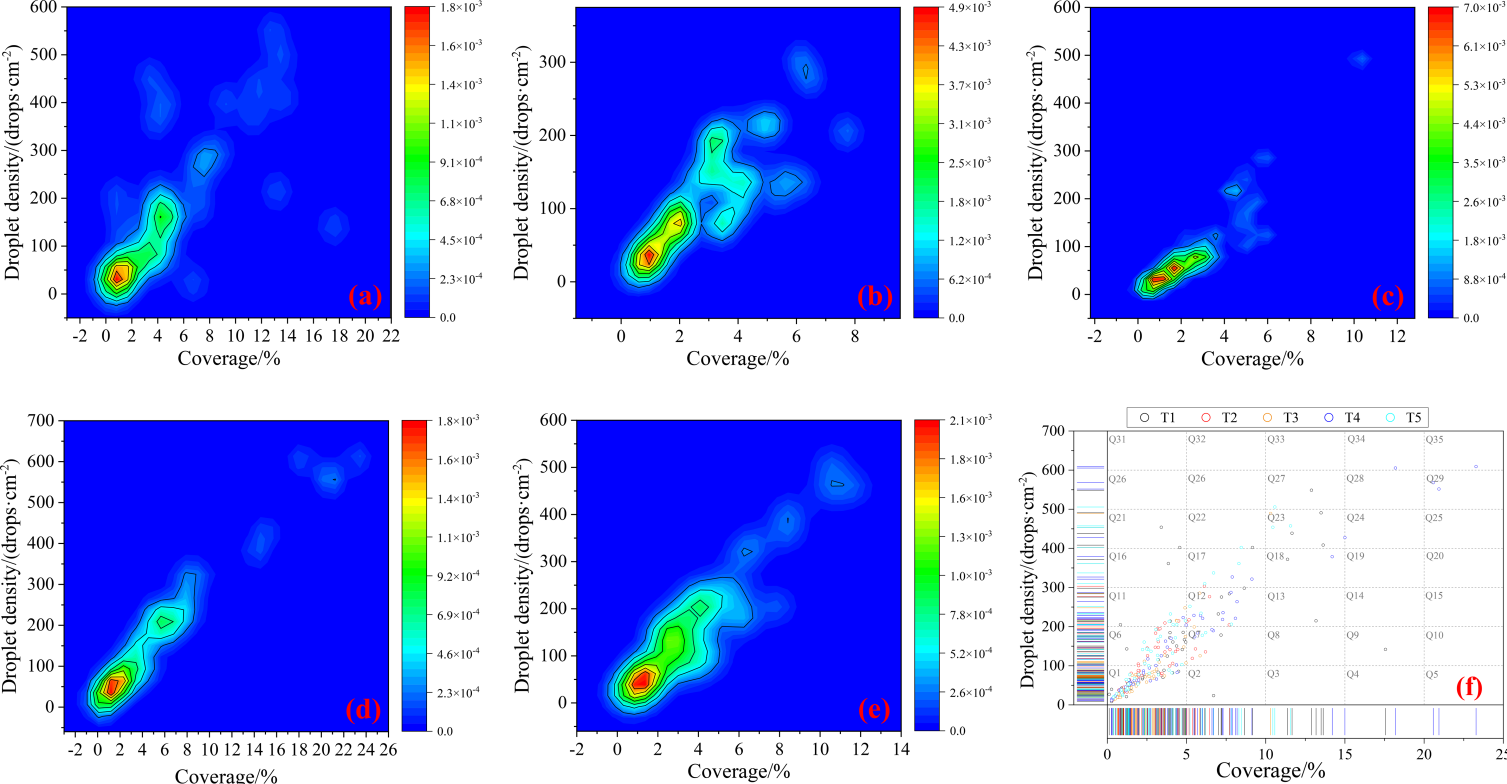
Statistics of droplet distribution ranges at sampling points for **(a)** T1; **(b)** T2; **(c)** T3; **(d)** T4; **(e)** T5; **(f)** overall summary.

The distribution of droplet coverage and droplet density at all sampling points for each treatment was statistically analyzed, as shown in **Figure 5**. **Figures 5a–e** display the droplet distribution for each treatment, with the horizontal axis representing coverage and the vertical axis representing droplet density. The different colors in the figures represent the quantity of droplet deposition at each location, with colors closer to red indicating a higher amount of deposition at the corresponding position. **Figure 5f** further summarizes the droplet deposition for each treatment in a scatter plot. The droplet coverage range (0-25%) was divided into five equal intervals, and the droplet density range (0–700 drops/cm²) was divided into seven equal intervals, resulting in 35 droplet distribution regions, labeled sequentially as Q1 to Q35.

It is evident that the droplets for all treatments are primarily concentrated in the Q1 (51.48%) and Q6 (20.37%) regions, meaning that 71.85% of the droplet coverage is in the 0-5% range and the droplet density is in the 0–200 drops·cm^-^² range, which aligns with the characteristics of the droplet distribution for Eavision UAV’s mist spraying operation. Further statistical analysis of the treatments reveals that the droplet proportions in the aforementioned regions (Q1 + Q6) for each treatment were 64.81% (T1), 81.48% (T2), 85.19% (T3), 59.26% (T4), and 68.52% (T5). Based on the spraying times settings in this study, it is evident that treatments with two spraying passes (T2 and T3) have significantly higher droplet proportions in these regions compared to the treatments with only one spraying pass (T1, T4, and T5). Additionally, the regions without droplets for each treatment was also calculated. The proportion of blank regions was 60% (T1), 82.86% (T2), 82.86% (T3), 68.57% (T4), and 74.29% (T5). The treatments with two spraying passes (T2 and T3) again exhibited significantly higher proportions of blank areas compared to the treatments with only one spraying pass (T1, T4, and T5). This further confirms that increasing the number of spraying passes has a positive effect on enhancing droplet aggregation, resulting in a more concentrated distribution of droplet coverage and density.

## 4 Discussion

After the pesticide is sprayed by the UAV, it needs to be settled on the target crop. Effectively improving spraying performance is an urgent need in the current development of UAV technology in crop protection. This study on flight patterns in mountainous orchards represents an innovative effort to address this challenge. Based on the results presented earlier, the outer canopy zone receives droplets more readily than the inner zone, primarily due to reduced obstruction, which increases the probability of droplet interception. Moreover, many studies have pointed out that the deposition effect on target crops varies depending on the level of disturbance from the rotor airflow (Guo et al., 2021). In this study, the disturbance of rotor airflow experienced by the tree canopy varied significantly across different treatments, with particularly notable differences among the flight patterns of T1, T4, and T5. Due to the influence of UAV rotor airflow and the constraints of the complex canopy structure of the trees, the deposition process of droplets released from the nozzles onto the leaf surfaces is highly random and uncertain. In the inter-row flight pattern, the rotor airflow mainly interacts with the canopy from the side, which makes it easier to observe higher droplet deposition in the middle and lower layers of the canopy. In contrast, when the UAV flies above the top of the fruit tree canopy, the rotor airflow develops vertically downward and disperses in all directions after contacting the canopy. The core airflow region spreading upward in the direction perpendicular to the tree rows collides with the vertical terrace surface of the orchard and then spreads along the ground. The airflow spreading downward in the direction perpendicular to the tree rows mainly impacts the canopy of the next row of fruit trees, while the airflow spreading along the tree-row direction mainly flows along the ground. The large distribution range of C_IO_ and C_D_ values in section 3.4, as well as the generally high CV-U values, further confirm this viewpoint. Therefore, although the coverage and droplet density in the outer zone of the canopy tend to be higher than in the inner zone, the differences in data are not statistically significant. This is also consistent with the complex and variable nature of field spraying trials (Chen et al., 2025).

There are also some differences in the distribution of droplets in the vertical direction of the canopy across different operational patterns. The EA-30X UAV used in this study is equipped with the CCMS misting nozzle, which produces a conical spray distribution. The horizontal cross-section of the spray expands progressively from top to bottom, enabling broader coverage of the fruit tree canopy and consequently increasing droplet deposition. When the UAV flight path is directly above the tree, the droplets directly impact the center of the canopy, making it easier for the canopy to be penetrated. This is the direct reason why the CV-P values for coverage rate and droplet density in T1 are 7.34% and 3.95%, respectively. When the flight path is between the tree rows, droplets primarily contact the outer zone of the canopy, leading to the greatest deposition differences between the inner and outer canopy zones (the C_IO_ value for T4 is much smaller than T1). Moreover, due to the droplet rebound effect upon hitting the ground (Shan et al., 2024), droplets are more likely to settle in the middle and lower layers of the canopy. When the flight path is perpendicular to the tree row, the flight path does not necessarily pass directly above the tree canopy. In T5, droplet deposition in the upper and middle layers is similar, while the lower layer shows significantly higher deposition. This could also be related to the droplet rebound effect. In this flight pattern, the UAV may alternately pass over the tree rows and the inter-row gaps, which can help improve the uniformity of deposition both the inner and outer canopy zones.

At the same time, the change in deposition effects caused by the increase of spraying passes (T2 and T3) should not be overlooked. From the statistical results, it is evident that compared to the treatments with only one spraying pass, T2 and T3 generally show lower droplet deposition in both the inner and outer canopy. In the vertical direction, both T2 and T3 show the lowest deposition in the upper layer, with a gradual increase in deposition in the middle and lower layers. Under the same spray volume rate for each treatment, this may be related to the two spraying passes performed in T2 and T3. When the UAV flies along the top of the tree for spraying, the downwash airflow from the rotors is very strong. Under the influence of this strong rotor airflow, the upper part of the canopy is somewhat opened (Sassu et al., 2024). During the second spraying, droplets can easily settle in the middle and lower parts of the canopy, thus significantly improving deposition in those layers. Further analysis also reveals that the deposition effect in T2 is overall better than in T3. This may be because T3 uses a smaller application rate (30 L/ha), which leads to a smaller flow rate in the spraying system and consequently lower pressure. As a result, the atomized droplets tend to have a larger diameter. Research has shown that smaller droplets are more likely to penetrate the gaps between the canopy leaves and reach various positions within the canopy (Wang et al., 2024). Therefore, the droplet deposition in T2 is higher. From another perspective, it can also be inferred that for flying patterns with increased spraying pass, changing the application rate per pass has a greater impact on droplet deposition than altering the operational speed. Additionally, the distribution analysis of sampling points in this study revealed that increasing the number of spray passes helps enhance the aggregation of deposited droplets, it’s a notable change and a fortuitous finding. However, this experiment only included one control treatment for spray volume and speed, the results may be influenced by chance, and the subtle differences in meteorological conditions during the operation of each treatment could also affect droplet deposition. For instance, the relatively high wind speed at T2 (1.3 m/s) may interact with the rotor airflow of the UAV to some extent, which could potentially explain the lower droplet deposition observed in T2. Future research should further improve the experimental design and conduct more detailed validation studies focusing on the impact of meteorological factors.

In general, T1 and T4 have similar overall droplet deposition, which are also the highest, but the deposition at different canopy heights differs between the two treatments. When the UAV flies directly above the tree rows, the droplet deposition in the upper layer is higher than in the middle and lower layers. However, when the UAV flies between the tree rows, the droplet deposition in the middle and lower layers is higher than in the upper layer. For T5, deposition was better in the lower than in the upper canopy, but its overall deposition was lower compared to T4. One advantage of T5 is that it improves the deposition uniformity between the inner and outer canopy zones. The droplet deposition distribution in T2 and T3 is similar, with the lower layers showing better deposition than the upper layer. However, the overall deposition for both remained low. Moreover, from the perspective of operational efficiency, applying two spray passes required a longer operation time. Hence, the flight patterns of T2 and T3 are not recommended. Given that this study was a preliminary exploration of the effects of UAV flight patterns on droplet deposition in Nanguo pear orchards, water was used instead of pesticides in the repeated experiments to avoid potential pesticide damage. Future studies should incorporate actual pesticide spraying operations and further expand research on environmental drift risks and pesticide residues on target crops. In addition, a comparison with the study by Wang et al. (2022) indicates that the conclusions regarding flight patterns in the present study appear to differ from theirs. However, such discrepancies actually reflect the differences in UAV applications between mountainous and flat orchards, which further highlights the necessity of conducting targeted experiments under different orchard types and operational environments.

Overall, this study represents a further extension of existing research on aerial spraying applications in mountainous orchards and, to some extent, fills the gap in studies on flight pattern selection for electric UAVs in such environments. It also helps operators make more flexible decisions when planning operational strategies and provides a useful reference for achieving more precise and efficient pest and disease control.

## 5 Conclusion

This study comprehensively measured and evaluated the droplet deposition effects of UAV aerial pesticide application under various flight patterns in a mountainous Nanguo pear orchard. With the total spray volume rate and flight height remaining constant, the two-pass UAV flight pattern (intra-row-high-speed and intra-row-half-rate) for orchard spraying is not recommended, especially when the natural wind speed is high. As it not only affects UAV operational efficiency but also results in unsatisfactory spraying effectiveness. But the study also confirmed that increasing the number of spray passes enhances droplet aggregation, resulting in more concentrated distributions of both coverage and density. Three flight patterns (intra-row, inter-row and verti-row) are viable options, but the choice should be made flexibly based on operational objectives. If higher deposition in the upper canopy is desired, intra-row should be selected; if higher deposition in the middle and lower canopy is desired, inter-row is the better option; and if more uniform deposition between the inner and outer zones is needed, verti-row is recommended.

## Data Availability

The raw data supporting the conclusions of this article will be made available by the authors, without undue reservation.
